# From 60 Causes to 10 Solutions Against Antibiotics Shortages in Sweden: Platinea’s Approach to Developing Policy Interventions

**DOI:** 10.3390/antibiotics14121249

**Published:** 2025-12-10

**Authors:** Enrico Baraldi, Håkan Hanberger, Sofia Wagrell

**Affiliations:** 1 Department of Business Studies, Uppsala University, 75120 Uppsala, Sweden; 2 Department of Biomedical and Clinical Sciences, Linköping University, 58183 Linköping, Sweden; hakan.hanberger@liu.se; 3 Department of Civil and Industrial Engineering, Uppsala University, 75237 Uppsala, Sweden; sofia.wagrell@angstrom.uu.se

**Keywords:** antibiotics, drug shortages, multi-sectoral collaboration, policy solutions, prioritization method

## Abstract

**Background/Objectives**: Antibiotic shortages are a growing problem which harms patient safety, increases healthcare costs, and accelerates antibiotic resistance. Based on the example of Sweden, the paper aims to illustrate and discuss how an organized collaboration platform can devise several policy interventions against shortages of antibiotics for human use. **Methods**: We describe how the multi-sectoral collaboration Platinea (Platform for Innovation of Existing Antibiotics) first identified, structured, and prioritized the causes of antibiotic shortages, and then identified and prioritized a set of policy solutions matching these causes. The specific methods applied include expert elicitation, interactive workshops, focus groups, and multi-criteria decision processes. **Results**: After an overview of about 60 causes of antibiotic shortages, we relate them to 10 prioritized solutions including, e.g., increased inventories, central coordination, integrated IT systems, increased unit prices, yearly fixed payments, and Nordic collaboration in purchasing and production. **Conclusions**: We propose a process with six steps to devise policy solutions by involving a multi-sectoral stakeholder group: open brainstorming of the problem’s causes, framing them into a clear structure, selecting and prioritizing key causes, matching causes and solutions, and devising prioritization mechanisms about emerging solutions. This approach can be applied to other national contexts and similar policy issues.

## 1. Introduction and Purpose

Less than 100 years after the discovery of penicillin, humanity is heading towards disastrous consequences due to escalating antimicrobial resistance (AMR), one of the largest threats to global health [1,2,3]. AMR stretches many intertwined problem areas, from overuse and misuse of antibiotics to regulatory, economic, production, and supply factors [4,5]. This paper addresses one such area of growing concern, namely shortages of antibiotics for human use, reflecting the general issue of drug shortages [6,7,8,9]. Antibiotic shortages have historically affected low- and middle-income countries, but are a global problem today, with severe effects also seen in high-income countries like Sweden [7,8,9]. This paper draws upon and discusses the work undertaken by a Swedish collaborative platform, Platinea, which was founded as an initiative to counteract antibiotic shortages and preserve the value of old antibiotics. Sweden constitutes an interesting example in the area of antibiotics: thanks to significantly lower AMR levels than in most countries, antibiotics that have lost their therapeutic effect in many parts of the world are still effectively used in Sweden [10]. Yet, a small market size combined with needs for unique products makes the Swedish antibiotic market especially vulnerable from a supply perspective [11].

The purpose of this paper is to illustrate and discuss how an organized collaboration platform can jointly develop several policy interventions against antibiotic shortages, exposing important links between the causes behind this problem and various policy tools, from specific stand-alone interventions (e.g., price increases) to complex interconnected ones (e.g., central coordination). In particular, we use the case of Platinea to illustrate how a structured methodology can be applied to jointly identify, via the contribution of parties from several sectors, first the single causes of antibiotic shortages and then specific solutions to address these causes. Hence, the Methods Section of this paper already features important results in terms of *how* policies can be developed in an *interactive* and *collaborative fashion*. Before entering into the detailed process followed by Platinea, we provide a brief overview of the general consequences and causes of antibiotic shortages.

The consequences of antibiotic shortages are multiple and often serious [12,13,14]. Lack of adequate antibiotics makes it more difficult to treat a specific infection or disease, causing side effects such as enhanced toxicity in treatment, high risk of malpractice, and large increases in healthcare spending [9,15,16,17]. The shortage of a specific antibiotic also implies that another suboptimal type of antibiotic has to be used instead, which may fuel resistance further [18]. The use of suboptimal antibiotics can also trigger yet another shortage due to unexpected increased use of the substitute drug, creating ‘cascade effects’ of shortages [19]. In addition, continuous shortage situations tend to elicit hoarding behavior in healthcare, which triggers even further shortages [20].

The causes of antibiotic shortages identified in policy reports and academic articles are numerous, complex, and occur at many levels [19,21,22,23,24,25,26]. To simplify this complex picture, the causes can be grouped into the following main areas: (1) *market-related issues*, including weak supply structures concentrated in a handful of Asian countries [27,28], low margins due to generic competition [29], price-focused public procurement procedures [30], short-term contracts without volume commitments [31], and irregular or declining sales volumes [32,33,34]; (2) *clinical use of antibiotics*, with a great variation in treatment praxis both between and within countries [35], because older antibiotics were developed with less encompassing clinical trials [36,37], which causes further fragmentation of already small markets, like the Nordics [38]; (3) *production-related issues*, as antibiotics often rely on outdated, inflexible facilities and experienced-based knowledge rather than formal documentations, which makes it difficult to transfer production between sites; (4) *regulations and institutional practices*, ranging from low transparency about supply chains [39] and their specific risks [18] to lack of communication and coordination among suppliers, buyers, healthcare, and public authorities, which may all hold essential information but do not share it with other actors [38].

In fact, improved collaboration between the various sectors involved in supplying and using antibiotics is already a solution per se against shortages [21] (pp. 15, 19) [26] (p. 55). Therefore, this paper presents how such collaboration can be manifested in tailored organizational structures like the Platinea platform. This serves as an example of multi-sectorial collaboration, which has also developed specific suggestions for interventions against antibiotic shortages, such as new reimbursement schemes, logistics arrangements, communication and coordination along supply chains, and purchasing routines. The paper is organized as follows: first, we introduce our methods, which corresponds with the procedure applied within Platinea, detailing how the members of this platform identified over 60 causes of antibiotics shortages and devised 35 possible policy solutions to address these causes; then we present our results, featuring the 10 most prioritized solutions, followed by a discussion of how the causes identified within Platinea relate with previous studies and how the 10 selected interventions intervene in antibiotic supply chains. We conclude by mentioning also the implementation status of some of these interventions in the Swedish context.

## 2. Methods

Platinea’s process of identifying solutions against antibiotic shortages in a Swedish context relied on expert knowledge elicitation [40]. In particular, the 25 or so experts we engaged represented different sectors (academia, with multiple disciplines, industry, healthcare, and public agencies). This offered the opportunity to include multiple and complementary knowledge domains. In practical terms, our methods consisted of a series of structured workshops, where discussion leaders exposed participants to a set of questions that were discussed interactively and repeatedly in small groups (4–6 people), similarly to a focus group approach [41]. Discussions followed prompts by the organizers, inviting experts to consider multiple criteria (e.g., short- vs. long-term effects, or economic vs. medical consequences) in both selecting and evaluating policy options for antibiotics [42]. We now provide more details of the context in which these structured discussions were conducted as well as the specific steps of our process.

Funded by Vinnova (the Swedish Innovation Agency), PLATINEA, i.e., the Swedish Platform for Innovation of Existing Antibiotics, was officially created in 2017 with the purpose of addressing, among others, the rising problem of antibiotic shortages in Sweden (www.platinea.se). After a formation period during which a restricted number of organizations from academia, industry, and healthcare defined Platinea’s goal and structure, the platform expanded in 2019 to include Swedish public authorities too. Today, Platinea includes a total of 22 organizations from the four sectors of academia, industry, healthcare, and public authorities [26] (p. 55). In the period of 2019–2020, a group of 16 organizations from these sectors intensively collaborated in order to (1) identify the causes of antibiotic shortages afflicting Sweden (a work which resulted in a complex map) and (2) devise solutions in the form of policy-driven interventions against shortages, a work which was later complemented by the selection of a restricted set of interventions to be prioritized according to the actors participating in Platinea.

**Figure 1:**
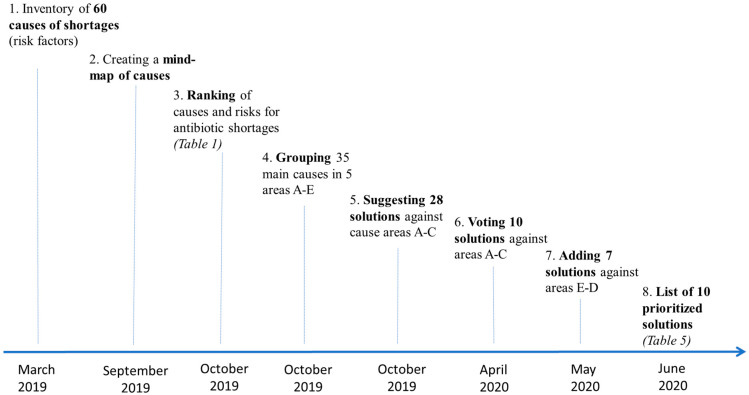
The process to generate Platinea’s 10 prioritized interventions against antibiotics shortages.

After introducing in Section 2.2 the 35 interventions against antibiotic shortages which the Platinea group initially identified, we shall focus on how 10 of these solutions were prioritized, considering also how each of them can influence the availability of antibiotics both in the short and the long term. Next to the various interventions, the Platinea group also identified and agreed on a key principle, which can be seen as the ground for many of the identified interventions: the need to clarify and make more visible the medical value of the key resource, namely antibiotics [43]. This section describes the methodological steps taken to first identify the potential causes of shortages and then prioritizes 10 interventions through a multi-disciplinary process and analysis, which involved around 25 participants engaged in Platinea work-package 4. Details on the 10 solutions are provided in separate reports (currently only in Swedish), available on Platinea’s website (e.g., https://www.platinea.se/download/18.4032e90218c8acf88513097/1703240487793/c_843338-l_3-k_platinea-delrapport-nr-6---central-koordiering-final.pdf, accessed on 8 September 2025). The entire process from identification of causes to selection of solutions was performed between March 2019 and June 2020 and included a total of eight steps, shown in Figure 1 below. The final list of selected solutions is provided in Section 3 (see Table 5 there).

In the following subsections, we describe the steps in Figure 1 starting from step 3, the ranking of causes of shortages, until step 8, the preparation of a list of 10 prioritized solutions. Details on the first two steps in Figure 1 are provided in other papers on Platinea’s website (https://www.platinea.se/w/pl/publikationer, accessed on 10 September 2025). Section 2.1 features how 60 causes of shortages were ranked and grouped into five thematic areas and how possible solutions were matched with each cause; while Section 2.2 describes how a total of 35 solutions were identified as ways to address the five areas of causes of shortages, including how the experts ranked them. The 10 prioritized interventions are then presented and discussed in Section 3 and Section 4.

### 2.1. Grouping 60 Causes of Antibiotic Shortages into Five Areas

**Figure 2:**
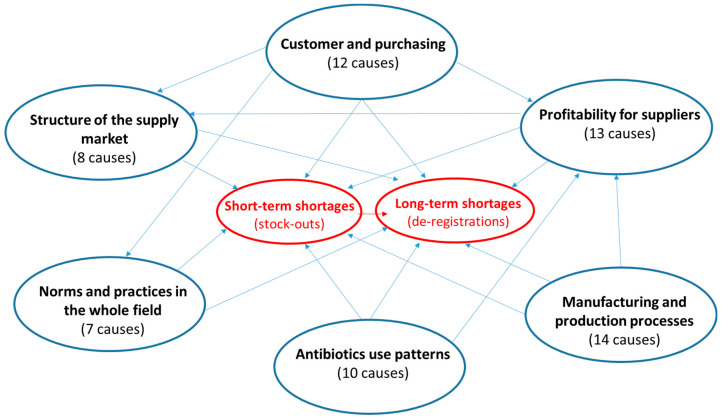
A simplified version of Platinea’s map of causes of antibiotic shortages.

Platinea had organized during 2018 and 2019 a series of discussions and workshops among about 25 participants from its parties representing academia, healthcare, industry, and public authorities, which resulted in a complex map displaying more than 60 causes of antibiotics shortages. Figure 2, seen below, shows a simplified version of this map (for the full picture see https://www.platinea.se/download/18.4032e90218c8acf8851313c/1703241238335/c_843338-l_3-k_platinea-orsakskarta-20200305.pdf, accessed on 8 September 2025).

Given the large number of causes (64 in total) that were identified through several brainstorming sessions, it was necessary to systematize them in order to obtain an overview of the problems afflicting the antibiotic sector. The first step in this systematization was grouping the causes depending on the type of stakeholder they originated from, e.g., customers (and their purchasing processes), suppliers, i.e., market authorization holders (MAHs), or producers, intended as manufacturers of finished dosage formulation (FDF). The map also identifies multiple and complex connections between shortages and various causes within each of the six categories shown in Figure 1. However, defining interventions based solely on the stakeholder (e.g., company or public actor) from which the shortage causes originate was considered by the facilitators of Platinea work-package 4 as an inadequate and potentially biased approach, because of two main reasons: (a) this approach would attribute the responsibility of causing shortages to specific stakeholders, whereas it appeared clear from most discussions that shortages depend on the coalescence of multiple causes from several categories of Figure 1, involving, as a consequence, many stakeholders at the same time; (b) this approach would also neglect the impact and relative importance of specific causes, irrespective of the stakeholder from which they originate.

Therefore, the next phase in the systematization of the causes of antibiotic shortages was to *rank and prioritize* the most serious causes. It was decided that only after this phase could causes be classified into groups to identify effective areas of intervention. We applied here a structured process to generate this prioritization, following three major steps:

(1) Platinea’s participants were invited to select the most important causes of antibiotic shortages among the 64 listed in Platinea’s complete map, according to the following voting mechanism: the 25 or so experts involved in Platinea’s work-package 4 belonged to three main types of organizations—academia, industry, and healthcare—and two persons were not formally linked to any organization in these three sectors. However, a majority of these experts belonged to the academic sector. Therefore, in order to avoid the bias of academic representatives favoring their own votes, it was necessary to allocate a maximum number of votes to each of the three sectors. In particular, to balance the views between Platinea’s sectors, individual experts were included in the following groups: four academic organizations, four industry-related organizations, three healthcare organizations, and two independent experts. Thus, all in all, 13 sets of votes were collected by email in reply to the following query: “Please, select up to 10 causes of antibiotic shortages that you consider as the most important among the ones presented in this list”.

The 35 causes of shortages (or factors that increase the risk of shortages) which received at least one vote in October 2019 are shown in Table 1 below here. Even if Platinea’s experts expressed a total of 130 single votes in selecting the most important causes of shortages, they eventually expressed only 113 votes, because some experts opted for selecting less than 10 causes among the most important ones. The four causes which were considered as the most important are “Low profit margins for market authorization holders (MAHs) and actors in the supply chain” (10 votes); “Reduced number of active pharmaceutical ingredient (API) suppliers” (9 votes); “Lack of volume commitments by buyers” (8 votes); and “Price-driven payment models” (6 votes).

(2) All causes which received at least one vote by Platinea experts were selected for further analysis. Moreover, one additional cause which was not included in the original map of causes was added during a teleconference meeting because several experts from multiple sectors pointed it out as important (see row nr 35 in Table 1). On the other hand, five causes of shortages, marked in red in Table 1, were excluded from further consideration because they appeared to be outside the scope of Platinea’s work-package 4 solutions, due to two main reasons: (a) they concerned the use of antibiotics rather than supply issues, or (b) they were indeed positive features of the Swedish market, which Platinea’s interventions should not change (e.g., the small size of the Swedish market with declining sales of antibiotics, the presence of many unique products with specific applications, or price competition between generics). Summing up, this left us with 30 causes of antibiotics shortages, which were used to generate solutions.

(3) We grouped the single prioritized causes into categories based on a common ground or root cause [44], because it appeared unfeasible to generate solutions for each one of the 30 prioritized causes in Table 1. This step led to the formation of, in November 2019, the following five core groups, each one relying on a root cause or key supply risk:

(A) Mismatch between the price and the actual value of antibiotics;

(B) Uncertainty;

(C) Inefficient logistics (physical and information flows);

(D) Lack of collaboration;

(E) Unbalances in the supply sector.

The single causes included in each group are shown in Table 2 below here, together with the potential solutions that were initially identified to address each single group of core causes, which will be considered in Section 2.2. The first two areas above (A and B) specify two root causes that are at the origin of “low profit margins” for several supply-related actors in the antibiotic field (i.e., the single most important cause according to Table 1), namely the fact that prices do not reflect the value of antibiotics (area A) and the uncertain or insufficient volumes that these supply actors can count on (area B). The other three groups of root causes indicate other types of deficiencies or unbalances in established structures that can cause lack of access to antibiotics: in the physical flows as well as the competence and information flows connected with logistics and production processes (area C), in the collaboration across the entire antibiotic field (area D), and in the structure and geographical location of the global supply sector (area E). Having created these five groups of causes, it was possible for Platinea experts to specify clusters of solutions that would together address each group of causes.

### 2.2. Identifying 35 Solutions Against the Causes of Shortages

To identify solutions against antibiotic shortages, Platinea work-package 4 organized first a brainstorming workshop with 25 participants in November 2019 and then a series of teleconferences until May 2020, covering solutions for specific areas of causes among the five presented above. In particular, the brainstorming exercise of November 2019 identified 28 solutions addressing areas A, B, and C, while the teleconferences helped devise seven additional solutions specifically for areas D and E. Thus, in total Platinea experts identified 35 solutions covering the five areas of causes of shortages. The logic that these experts followed to identify these solutions was that *each proposed solution* should address *at least one cause* among those listed under each single area, A–B–C–D–E (see Table 2, first column).

However, experts identified some solutions which addressed several causes of shortages at the same time, even causes belonging to more than one of the five areas. For instance, the solution “Yearly fixed payment for generic antibiotics” (nr 11 in the second column of Table 2) can address five causes belonging to two different areas. These five causes are as follows: (11) extreme focus by the public system on competitive bidding and market mechanisms, (23) difficult to apply other requirements than price and (24) margins on antibiotics are lower than other drugs (which belong to area A, “Mismatch between price and actual value of antibiotics”), and (3) lack of volume commitments by buyers and (25) uncertain demand (which belong to area B, “Uncertainty”). Similarly, the solution “Security stocks” can address six causes belonging to two areas: (25) uncertain demand and (33) unexpected volume changes in production/supply (which belong to area B, “Uncertainty”), and (8) insufficient communication healthcare–pharmacies–suppliers–authorities, (14) lack of security stocks, (18) rigid production systems and (35) limited planning ability of drug suppliers (which belong to area C, “Inefficient logistics”).

The various solutions were suggested by experts representing all sectors involved in Platinea’s brainstorming, namely academia, healthcare and industry. Public authorities were excluded from these specific steps (5–8 in Figure 1), because they were expected to be the recipient of Platinea’s policy suggestions. There was no requirement for consensus among experts to bring a solution to further scrutiny. However, since the initial brainstorming in November 2019 already generated many solutions (twenty-eight as per Table 2, and seven more were identified in the Spring of 2020), it would have been impossible to analyze all of them in detail. Therefore, as a way to identify a shortlist of solutions, in April 2020, Platinea’s experts were invited to vote to define a ranking of the strongest solutions among the 28 addressing the three areas, A, B, and C (see Table 2, right column). Similarly to the ranking of the causes of shortages, voting for the most relevant solutions involved four parties for each of the three sectors, namely academia, healthcare, and industry, and two independent experts (resulting in 14 voters in total, i.e., one more voter than for ranking causes, because this time healthcare organizations could also mobilize four voters). Each of the 14 voters was asked to rank the 10 most powerful solutions based on how well each one could address the causes of shortages listed in Table 2. Table 3 below shows the ranking and total votes for the solutions.

The solution which was ranked highest was “Security stocks, national and local/regional” (nr 12 in Table 3), with a total of 101 points, followed by various forms of increased central coordination in need planning, tendering and purchasing (nr 13 and 14 in Table 3). The 10th highest-ranked solution was “Increased max prices in auction system for products with very few MAHs”, with a total of 30 points.

This first ranking concerned the solutions addressing the first three areas—A (Mismatch between price and actual value of antibiotics), B (Uncertainty) and C (Inefficient logistics). However, the 28 solutions on Table 3 might or might not address the two remaining areas—D (Lack of collaboration) and E (Unbalances in the supply sector). Therefore, all experts in Platinea’s work-package 4 were involved in further discussions in May 2020 in order to identify new solutions, beyond the identified ones, which could address particularly well the lack of collaboration (area D) and unbalances in the supply sector (area E). During two teleconferences, the seven new solutions in Table 4 were identified. All participating experts considered four of these new solutions so closely related to the 10 highest-ranked solutions shown in Table 3, including them as components of some of these 10 solutions. Moreover, the experts considered three of the new solutions so important that they were added directly to the solutions that Platinea should eventually prioritize (see bold-text solutions in Table 4).

More specifically, three of the new solutions (nr 29, 30 and 31 in Table 4) concern mainly information exchange and management. Therefore, they were considered as being part of the “Integrated computer system” in solution nr 15 (Table 3). These three solutions (“Information exchange between suppliers”; “Enhanced communication required by the MPA” and “User-friendly tool for purchasers to identify bottlenecks”) can in fact be functions or components of this IT system, which can in turn be the computer-based cockpit for the national “master planner” expected to coordinate antibiotic supply among Sweden’s 21 regions (i.e., solution nr 21 in Table 3). The solution “Monetary incentives and preferred selection of MAHs who register multiple final dosage (FDF) and API suppliers” addresses area E (Unbalances in the supply sector) by reducing dependence on single suppliers and reinforcing delivery precision (solution nr 8 in Table 3). Moreover, these types of incentives and selection criteria can be part of the evaluation process for public tenders within the “Norwegian model” (solution nr 10 in Table 3).

As for the other three new solutions, “Nordic collaboration for purchase and production of older antibiotics” can reduce supply risks in two areas of causes of shortages: “Lack of (international) collaboration” (area D) and “Unbalances in the supply sector” (area E). Therefore, this solution was considered very important and added to Platinea’s prioritized solutions. The need to address the imbalances in supply (area E) was considered as so important that the last two additional solutions were devised by the experts specifically for this purpose. Both of these last solutions focus on regulatory processes: facilitating the introduction to Sweden of products already registered in other countries via “simplified regulatory processes” (nr 34 on Table 4) and avoiding the sudden disappearance of key products via “MAHs supporting orderly withdrawal” (nr 35 on Table 4). Orderly withdrawal would include the identification of alternative MAHs and the transfer of relevant documentation. Both of these last solutions were considered very important and were to be added to Platinea’s final list of 10 prioritized interventions against shortages, which is presented in the next section.

## 3. Results: Selecting 10 Prioritized Solutions to Reduce Antibiotic Shortages in Sweden

By combining and merging the 10 highest ranked solutions addressing areas A, B and C (Table 3) with the seven additional solutions addressing areas D and E (Table 4), the experts in Platinea work-package 4 eventually selected in June 2020 a final list of 10 prioritized interventions, which address all five areas of root causes and risks of antibiotic shortages. Table 5 below shows these 10 prioritized solutions. Moreover, these experts chose to append to the list also the core idea of “Clarify the medical value of antibiotics” (point 0). This was indeed one of the highest ranked solutions, coming up to eighth place in the ranking of Table 3. However, clarifying the value of antibiotics is such an abstract solution that the experts preferred to consider it as a general principle to motivate all other solutions, instead of considering it as an intervention per se. Yet, the experts also reckoned that the *introduction of new valuation models*, within the frame of health technology assessment (HTA) regulations and practices, can be a concrete intervention that can lead to allocating greater funds to antibiotics in reimbursement and hospital accounting systems [9,26]. Still, considering the complexities of HTA systems, Platinea experts preferred to reserve this type of intervention concerning the value of antibiotics for future discussion.

Looking specifically at how the single solutions have been selected and phrased, the solutions from nr 1 to 7 in Table 5 are taken directly from the 10 highest-ranked solutions addressing areas A, B, and C in Table 3. However, solution nr 2 in Table 5 below—“Central coordination with a master planner”—combines all three solutions concerning centralization listed in Table 3 (solutions nr 13, 14, and 21). As mentioned, the last three solutions in Table 5 are new solutions added to specifically address areas D and E (see Table 4) and were considered so important that they had to be included among the 10 most relevant interventions (the remaining four additional solutions for areas D and E in Table 4 were instead integrated as functions or components of solution nr 3, “Integrated computer system”, or solution nr 4, the “Norwegian model” in Table 5). 

These 10 prioritized interventions should be applied first of all to *critical* antibiotics, namely those with highest medical value and risking poor availability in Sweden according to the ranking published in three occasions since 2017 by the Public Health Agency of Sweden (PHAS) and featuring between 30 and 40 of such antibiotics [45].

When selecting these 10 interventions, Platinea’s experts chose explicitly not to rank them in terms of relative importance. Therefore, the numbers from 1 to 10 in Table 5 do not indicate any further order of prioritization. Instead, the numbers indicate a possible way to group the 10 solutions in terms of their focus and expected effect in the antibiotic field related to Sweden. In particular, solutions nr 1, 2, and 3 concern and require various forms of *central coordination* in the context of the Swedish healthcare system, which is based on 21 separate regions independently responsible for care provision [46]. Solutions nr 4 and 5 concern primarily *regional purchasing and reimbursement models*, even if the Norwegian model can, at least partly, be applied also to the national selection and auctions mechanism (see point 4.2 in Table 5), while solutions nr 6 and 7 are strictly *national reimbursement models*. Solutions nr 8 and 9 concern *regulatory processes* to simplify registration in Sweden and, respectively, plan and avoid sudden deregistration from Sweden. Finally, solution nr 10 embraces *international coordination* stretching outside Sweden. The various foci of the 10 solutions are discussed further in the next section, considering their role in a typical antibiotic supply chain.

## 4. Discussion

We discuss now our findings by considering first how the causes identified through Platinea’s structured approach relate with those found in the literature (Section 4.1) and then how the prioritized solutions intervene in antibiotics’ supply chains, indicating also their implementation status in Sweden (Section 4.2).

### 4.1. Relating Causes of Antibiotic Shortages Identified in This Study to the Literature

The causes of shortages that Platinea experts identified under the categories A and B, i.e., Mismatch price value and Uncertainty, correspond largely to those that the literature refers to as market conditions on the demand side, such as the following causes: low price of antibiotics because most products are old and exposed to generic competition for decades [29,47]; regulations imposing price-focused public tenders [30]; short-term contracts without volume commitments and unexpected volume variations with short-term notice [31]; continuously declining sales volumes [34]. Taken together, these causes induced several suppliers to leave some markets or even terminate production altogether due to low profitability [32,33]. Compared to previous studies that focused on single specific market-related causes, the systematic work within Platinea has the merit of relating them to the two root causes of low unit margin per product, summarized in the mismatch between value and price, and of uncertain (or low) volumes for whole categories of products. Identifying two separate root causes also allows the pinpointing of specific policy solutions that address the core problems related to *low unit margins* and *declining total volumes*. Despite diminished or uncertain use and low prices, from a medical point of view, it is imperative to maintain a wide array of different products and formulations available so doctors can switch among them and thereby contain resistance levels and secure optimal treatment for patients.

Many, but not all, of the causes identified by Platinea experts under category C, Inefficient logistics, are considered in the extant literature. Several reports identify *limited stocks* combined with an *inability to match fluctuating demand* as important causes of shortages [21,48]. Therefore, increased inventories (corresponding to Platinea’s solution nr 1, Table 5) are also proposed in the literature as a key solution against at least short-term stock-outs [24]. Unsurprisingly this is the policy intervention which was ranked highest also by Platinea’s experts (see Table 3). *Insufficient communication* between actors in the supply chain (MAHs, pharmacies, healthcare) and public authorities is a cause that Platinea experts included both in category C, Inefficient logistics, and D, Lack of collaboration, because of its dual negative effect on both physical flows and coordination possibilities, as also reckoned in the literature [18,49]. Within category C and its focus on physical issues, Platinea experts identified (by combining technical and regulatory limitations) a series of causes that the literature has *not* recognized, namely inflexible and outdated factories, with production processes based more on experience-based knowledge than formal documentation, which makes it *difficult to transfer production to better factories*.

As for the causes of shortages in category D, Lack of collaboration, they also appear in the literature about issues in regulatory and institutional practices. Payors and other actors on the ‘demand side’ lack understanding of the complexity in the supply system of antibiotics [18]. There is also a great variation in treatment praxis both within the same country, e.g., Sweden, and between countries with similar epidemiology, e.g., the Nordics [35,50], because old antibiotics still in use today were approved before the detailed usage instructions of modern clinical trials [36,37]: this lack of collaboration between healthcare professionals in defining common needs further fragments already small markets [51]. Another example of lack of collaboration is that authorities often fail to share key information about suppliers with healthcare providers, due to missing communication pathways or legal constraints [38]. Therefore, public actors are forced to handle shortages locally as they occur, lacking both an overview of risks over their supply networks and in-depth understanding of the shortage problem. This issue is related to the *lack of transparency* across supply chains [39], which is another major barrier to preventing and handling shortage occurrences. Yet, it has proven difficult to improve transparency about complex supply networks with several sub-suppliers located all over the globe, because the pharmaceutical industry is highly competitive and relies on strict legal protection of what they perceive as highly confidential information about their supply chains [49].

Finally, the causes identified by Platinea experts under category E, Unbalances in supply, are commonly discussed in the literature as supply issues. In particular, the global supply market today is concentrated in a handful Asian countries, as a consequence of heavy outsourcing to low-cost countries during the 1990s [27]. This extensive outsourcing and consolidation of the supply market has resulted in *weak supply chains* [9], which are sensitive even to minor disturbances; and this has provoked a situation where many Western countries are dependent on single offshore suppliers for essential drugs [28], which entails enhanced geo-political and disaster risks. One of the most commonly reported causes of antibiotic shortages is the disruption in raw material production leading to API scarcity [21], while one of the world’s largest API-producing countries, India, has reported a decline in API production over the years [52].

### 4.2. How Do the 10 Prioritized Solutions Intervene in Antibiotic Supply Chains?

**Figure 3:**
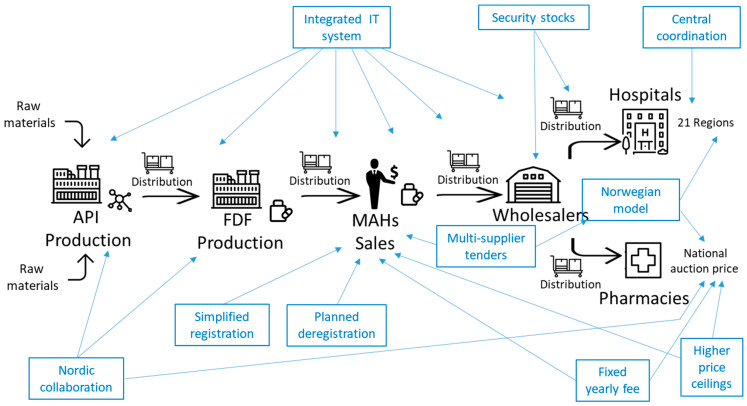
The expected roles of the 10 prioritized solutions in the Swedish antibiotic supply chain. Source: Baraldi et al. [28] (p. 625).

As shown in Section 3 and Section 4 and Table 2, the 10 solutions selected in Table 5 address different causes of shortages. These causes were grouped by Platinea experts into five categories (A-E, see Table 2) and can in turn be associated with one or more actors involved in *antibiotic supply chains*. Platinea experts indeed represented these various actors, shown in Figure 3: API (Active Pharmaceutical Ingredient) suppliers; FDF (Final Dosage Formulations) producers; market authorization holders (MAHs); wholesalers and hospitals or pharmacies. As Platinea’s 10 prioritized solutions address different root causes and the associated actors, we can now discuss how each one of these solutions is expected to intervene in different parts of the antibiotic supply chains related to the Swedish market (see Figure 3). We also consider the support found in the literature for each solution, even if most studies were published *after* Platinea prioritized these solutions (in June 2020), as well as which solutions have been or are being implemented in Sweden at the time of our writing (November 2025).

The “integrated IT system” (nr 3 in Table 5) is the solution that we expect to intervene most broadly *across the whole supply chain* because it aims to improve the connections and information flows between all links of the chain [53] (p. 221), including the overview of the whole process and available stocks, which is necessary to balance needs and delivered volumes, as proposed also at the international level by the European Commission [24] (pp. 13–14) and Shafiq et al. [9] (p. 5). By contrast, “Nordic collaboration” (nr 10 in Table 5) acts *either upstream or downstream* in the chain. This solution, suggested also in an official report by three key Swedish authorities [54] and elaborated by Baraldi and Wagrell [38], intervenes *upstream* by improving how the Swedish market connects with API and FDF producers, typically located outside Scandinavia [28]. Moreover, “Nordic collaboration” intervenes *downstream* in the chain by rationalizing the range of products used in Swedish healthcare in relation to those used in neighboring countries [51]. Current discussions among the Nordic countries actually concern collaboration on joint procurement and potentially also on production, following especially the recent acquisition by the Swedish government of an antibiotic factory previously owned by one of Platinea’s partners [55].

“Security stocks” (nr 1 in Table 5) is a core proposal in many studies [24,56] (p. 5) and may affect any level of the supply chain, because additional inventories can be created in any link of the chain. However, Platinea’s proposal concerns the *wholesale level* of the chain. In fact, in January 2020, Platinea experts proposed to the Swedish government to increase the volume of available inventories, specifically at the only two nation-wide wholesalers, because these constitute the link in the Swedish supply chain where nationally coordinated stocks can be held and managed more swiftly [53] (p. 211). Currently, the Swedish government has mandated in 2025 two central health-related authorities to define a model for holding increased stocks of specific medicines [57].

These stocks also relate to the next solution, “central coordination” (nr 2 in Table 5). In fact, centralizing, specifically stockholding and procurement, is an intervention against drug shortages proposed in the literature also by Ahlqvist et al. [53] (pp. 219–221). More broadly and in the EU context, the Technopolis Group [48] (p. 89) stresses the importance of centralized coordination. In the specific Swedish context, this intervention builds on several tools acting *mostly downstream* in the supply chain, that is, on processes performed at the regional level: these tools include monitoring and assembling the needs of all Sweden’s 21 regions, as well as coordinating their antibiotic purchases and distribution, which normally occur starting from each single region. Another solution that involves the *regional downstream level* of the chain (i.e., regional purchases for hospitals), but connects it with the *level of MAHs*, is “Spreading supply volumes through multi-supplier tenders” (nr 5 in Table 5). This solution reflects the logic of multiple-sourcing promoted for the EU level also by Panteli et al. [56] (pp. 3–5) and the Technopolis Group [48] (pp. 98–99). Another solution that starts from *customers downstream in the chain*, both at the regional and the national level in Sweden, is the “Norwegian model” (nr 4 in Table 5). This model owes its name to the fact that Norway required, earlier on, to purchase drugs by using the “Most Advantageous Economic Tender” (MEAT) criteria, i.e., criteria beyond unit prices. This practice is advocated in the literature by Panteli et al. [56] (pp. 2–4) and is also required in the proposal for the EU’s Critical Medicines Act [58] (pp. 12, 22).

Finally, there are four interventions which *act specifically on MAHs at the national level*, although in different ways. “Increased max prices in national auctions” (nr 6 in Table 5) reflects the widely recognized need to raise prices for generic antibiotics [54,59]. This solution increases revenues for any MAHs supplying products with no or very few alternative MAHs, by allowing the remaining MAHs to have higher price ceilings and thus unit margins on their product. Eventually this Platinea solution was implemented by Sweden’s Dental and Pharmaceutical Benefits Agency in 2023 [60]. A “fixed yearly fee” (nr 7 in Table 5) creates certainty for MAHs by providing a guaranteed minimum payment every year, with contracts similar to a subscription [56] (p. 4) [59]. Even if not yet implemented, in 2024, the Public Health Agency of Sweden proposed a model for a yearly fixed payment specifically for 20 selected generic antibiotics [61].

While increased prices and fixed yearly payments are solutions that affect MAHs through reimbursement, the next two solutions concern registration processes: “simplified registration” (nr 8 on Table 5) allows MAHs to save costs and time to start selling more swiftly an antibiotic new for Sweden, but already approved in other countries [26] (p. 51); “planned deregistration” (nr 9 on Table 5) entails that a new MAH takes over an existing product which its current MAH would withdraw from the Swedish market [54] (pp. 137–138). Even if the 10 solutions address specific parts of the antibiotic supply chain, they are also connected and support each other. For instance, “central coordination” implies that a master planner employs some kind of IT-based tool, like the “integrated IT system”, which is in turn a key tool to calculate and monitor the levels of “security stocks”.

## 5. Conclusions

This paper has described the systematic approach to identify 10 selected interventions against antibiotic shortages, devised and applied by the multi-sectorial collaboration platform Platinea. A formal recognition of the relevance of these 10 solutions came in September 2021 when the Swedish Government officially mandated three main central public authorities to assess the possibility to implement Platinea’s suggestions [62], resulting in a detailed mapping that summarizes this governmental mission [54]. Moreover, as mentioned in Section 4, four of Platinea’s policy solutions (security stocks, higher prices, yearly fixed payments, and Nordic collaboration) have been implemented or are the object of dedicated pre-studies by the relevant public authorities. While we leave it to other studies to penetrate the details of single interventions, this paper focused on describing the *methodological process* which led various stakeholders to identify and collaboratively select these solutions. The key lessons from this process, which can be applied to similar policy-oriented tasks requiring multi-sectoral collaboration, are as follows:

1. The importance of *brainstorming openly* the many causes of the problem at hand, exploiting the domain-specific competence and unique vantage point of each single stakeholder;
2. Despite this initial openness, it is then necessary to create *an ordered structure* for the numerous causes identified, as a step to start generating again, via brainstorming, the possible solutions (point 4 below);
3. If the causes identified in step (1) are too many, it is necessary to identify the key ones and *prioritize the most serious causes* as a way to further guide the selection of solutions/policy interventions;
4. Despite the usefulness of starting with open brainstorming for solutions, it is necessary to balance this open, explorative process by *matching solutions* as clearly as possible *to specific causes* of the problem;
5. Due to the wealth of solutions likely proposed by a heterogeneous multi-sectoral group of experts/stakeholders, it is necessary to *devise a commonly accepted selection mechanism* that makes everyone feel involved: for instance, a particular voting mechanism with implicit ranking and points attributed, similar to multi-decision criteria analysis [42];
6. Loop back the selected solutions to the original causes, although this time the causes should be expressed in different terms, e.g. the graphical display of a supply chain. This loop and alternative matching between solutions and causes may indicate specific areas/points where interventions are lacking and need to be elaborated. In more general terms, it is helpful to anchor the solutions to theory and established models over the system which the solutions aim to affect [43].

A limitation of this study concerns the highly context-specific nature of its findings (the identified causes and selected solutions) and partly of the methods applied. A different national context and group of stakeholders would have likely identified and prioritized different causes and solutions. A more general methodological limitation is that the selection and voting mechanisms involving a heterogenous group may dilute via average scores the impact of some causes and solutions which may instead be very urgent for a single actor. Further research should be dedicated to elaborating the details of single interventions, their pros and cons, as well as to discussing how they can be implemented in the specific Swedish context and in other national contexts.

## Figures and Tables

**Table 1:** Platinea’s prioritized causes of antibiotic shortages (October 2019).

Cause of Antibiotic Shortages	Votes (113 Total)
(1) Low profit margins for market authorization holders (MAHs) and actors in the supply chain	10
(2) Reduced number of active pharmaceutical ingredient (API) suppliers	9
(3) Lack of volume commitments by buyers	8
(4) Price-driven payment models	6
(5) Inappropriate use of antibiotics: broad spectrum use reduces sales of narrow spectrum	5
(6) Dependence on too few suppliers and MAHs with low profitability	5
(7) Sweden is a market with small antibiotic usage volumes	5
(8) Insufficient communication amongst healthcare–pharmacies–suppliers–authorities when stock-outs occur	4
(9) Limited ability to pay by the public healthcare system	4
(10) The public management system does not grasp the complexity in supply chains and their risks	4
(11) Extreme focus by the public system on competitive bidding and market mechanisms	4
(12) Low and decreasing sales volumes	4
(13) Many unique products with small sales volumes, hard to produce and handle all of them	4
(14) Lack of security stocks	3
(15) Price-based competition among generics	3
(16) Older factory, too expensive to upgrade to new requirements and standards	3
(17) Harder environmental requirements	3
(18) Rigid production systems	2
(19) Decreased number of European producers	2
(20) Antibiotics are a low-priced product	2
(21) Decentralized (regional) purchasing fragments the market	2
(22) Factories concentrated in just a few countries	2
(23) Difficult to apply other requirements apart from price, e.g., delivery precision or sustainability	2
(24) Margins on antibiotics are lower than on other drugs	2
(25) Uncertain demand	2
(26) Weak cooperation among organizations in the whole system	2
(27) Lack of partnership in Scandinavia and Europe in improving access to old antibiotics	2
(28) Lack of transparency in supply chains	2
(29) Risk of natural catastrophes	2
(30) Geo-political risk (trade conflicts, etc.)	1
(31) Difficult to transfer old production permits/certifications (files) between factories	1
(32) Old products with hard to update files (permits)	1
(33) Unexpected volume changes in production and supply	1
(34) The Swedish open auction for monthly price setting (“Periodens vara-system”)	1
(35) Limited planning ability of drug suppliers (*added after additional teleconference meeting*)	Oral votes

**Table 2:** Causes of shortages grouped in five areas and possible solutions (November 2019 and May 2020).

Area (in Bold) and Single Causes of Shortages	Possible Solutions
**(A) Mismatch between price and actual value of antibiotics**	1. Clarify the medical value of antibiotics 2. Cross-subsidize antibiotics with high-profit products at pharmacies 3. Reallocate healthcare budgets to favor antibiotics 4. Tenders or auctions with multiple winners 5. Spread supply volumes among several suppliers 6. Increase max prices in auction system for products with very few MAHs 7. Sustainability/environment reward 8. Delivery precision reward 9. Assortment breadth reward (holding many antibiotics) 10. Norwegian model combining 7 and 8 11. Yearly fixed payment for generic antibiotics
(9) Limited ability to pay by public healthcare system	
(4) Price-driven payment models	
(11) Extreme focus by the public system on competitive bidding and market mechanisms	
(20) Antibiotics are a low-priced product	
(23) Difficult to apply other requirements apart from price, e.g., delivery precision or sustainability	
(24) Margins on antibiotics are lower than other drugs	
**(B) Uncertainty**	4. See above 11. See above 12. Security stocks 13. Central coordination: need planning and prioritization 14. Centralized tendering and purchasing 15. Integrated computer system: AI balances needs and supplies 16. Logistics competence sourced from sectors familiar with uncertain demand 17. Extend from monthly to trimestral auctions
(3) Lack of volume commitments by buyers	
(21) Decentralized purchasing fragments the market	
(25) Uncertain demand	
(33) Unexpected volume changes in production/supply	
(34) The Swedish open auction for monthly price setting	
**(C) Inefficient logistics (physical and information flows)**	12. See above 15. See above 18. IT system with early warnings and incident reporting 19. Fining MAHs who miss reporting stock-outs 20. MAH must report delivery precision for several years 21. One national master-planner responsible for drug availability 22. Harmonized packages between countries 23. Prolonged shelf-life (expiry dates, grant exceptions) 24. Knowledge sharing between factories and companies 25. Moving generics production to modern factories closer to Sweden 26. Regulatory relief to transfer old products to new factories 27. Environmental subsidies for technology upgrades 28. Industry associations-led training on demand forecast for pharma personnel
(8) Insufficient communication amongst healthcare–pharmacies–suppliers–authorities when stock-outs occur	
(14) Lack of security stocks	
(16) Older factory (too expensive to upgrade to new requirements and standards)	
(17) Harder environmental requirements	
(18) Rigid production systems	
(31) Difficult to transfer old production permits/certifications (files) between factories	
(32) Old products with hard to update files (permits)	
(35) Limited planning ability of suppliers	
**(D) Lack of collaboration**	29. Information exchange between suppliers in case of stock-outs: database with clearing mechanism 30. Stricter requirements to the Medical Products Agency in case of stock-outs (tex. proposing alternative MAH) 31. Computer-based system for purchasers to identify bottlenecks and risks in supply chains 32. Nordic (or EU-level) collaboration for purchase and production of older, narrow-spectrum antibiotics
(8) Insufficient communication amongst healthcare–pharmacies–suppliers–authorities when stock-outs occur	
(10) The public management system does not grasp the complexity in supply chains and their risks	
(26) Weak cooperation in the whole system	
(27) Lack of partnership in Scandinavia and Europe to improve access to old antibiotics	
(28) Lack of transparency in supply chains	
**(E) Unbalances in the supply sector**	32. See above 33. Rewarding MAHs who register several FDF (final dosage) and API suppliers, from several countries. 34. Regulatory simplifications to register in Sweden older antibiotics approved in other countries 35. MAH supporting ordered product withdrawals via alternative producer/MAH
(2) Reduced number of API suppliers	
(6) Dependence on too few suppliers and MAHs with low profitability	
(19) Decreased number of European producers	
(22) Factories concentrated in just a few countries	
(29) Risk of natural catastrophes	
(30) Geo-political risk (trade conflicts, etc.)	

**Table 3:** List and ranking of 28 solutions addressing areas, A, B, and C (April 2020).

	Overall Rank (1–10) *	Total Votes **
**1.** **Clarify the medical value of antibiotics**	**8**	42
2. Cross-subsidize antibiotics with high profit products at pharmacies		
3. Reallocate healthcare budgets to favor antibiotics		4
4. Tenders or auctions with multiple winners		20
**5.** **Spread supply volumes among several suppliers**	**4**	63
**6.** **Increase max prices in auction system for products with very few MAHs**	**10**	30
7. Sustainability/environment reward (or tendering requirement/criterion)		13
8. Delivery precision reward (or tendering requirement/criterion)		9
9. Assortment breadth reward (holding many antibiotics)		24
**10. Norwegian model combining 7 and 8 (supplier selection based on environmentally sustainable and precise supply)**	**5**	61
**11 Yearly fixed payment for generic antibiotics with volume commitments**	**9**	40
**12. Security stocks, national and local/regional**	**1**	101
**13. Central coordination: need planning (from local to national level) and prioritization during shortages**	**2**	75
**14. Centralized tendering and purchasing (or joining multiple regions), but awarding multiple suppliers**	**3**	74
**15. Integrated computer system with overview on stock levels: AI balances needs and supplies**	**7**	42
**16. Logistics competence sourced from sectors familiar with uncertain demand (e.g., retailing, fast-moving consumer goods)**		7
17. Extend from monthly to trimestral auctions (or even longer for products with few available suppliers)		8
18. IT system with early warnings and incident reporting		14
19. Fining MAHs who miss to report stock-outs		10
20. MAH must report delivery precision for several years		6
**21. One national master planner responsible for drug availability (a stronger version of nr 13)**	**6**	44
22. Harmonized packages between countries		7
23. Prolonged shelf-life (extended expiry dates, grant exceptions)		24
24. Knowledge sharing between factories and companies		
25. Moving generics production to modern factories closer to Sweden		23
26. Regulatory relief to transfer old products to new factories		9
27. Environmental subsidies for technology upgrades		11
28. Industry associations-led training on demand forecast for pharma personnel		

* Bold text shows the 10 highest ranked solutions. ** Each expert attributed 10 points to the solutions considered as most important and 1 point to the least important among the 10 selected ones.

**Table 4:** Seven new solutions addressing areas D and E (May 2020).

	Note (Links to Table 3)
29. Information exchange between suppliers in case of shortages (“clearing mechanism”)	Can be part of the master-planner’s “IT cockpit” with AI (nr 15, 21)
30. Enhanced communication required by the Medical Product Agency (MPA) in case of shortages (e.g., suggestion of alternative MAHs)	Can be part of the master-planner’s “IT cockpit” with AI (nr 15, 21)
31. User-friendly tool for purchasers to identify bottlenecks and risks in supply chains	Can be part of the master-planner’s “IT cockpit” with AI (nr 15, 21)
**32. Nordic collaboration for purchase and production of older antibiotics**, esp. narrow-spectrum child formulations, e.g., rationalized and optimized product ranges, harmonized pack sizes in the Nordic countries	Addresses both area D and E. Added as a new solution to be prioritized
33. Monetary incentives and preferred selection of MAHs who register multiple final dosage (FDF) and API suppliers, ideally from several countries	Can be a measure of delivery precision (nr 8) or part of the Norwegian model (nr 10)
**34. Simplified regulatory processes** to introduce to Sweden old antibiotics approved in other countries	Addresses mainly area E. Added as a new solution to be prioritized
**35. MAHs supporting orderly withdrawals** with substitute supplier/MAH (incentive to transfer authorizations/product registrations)	Addresses mainly area E. Added as a new solution to be prioritized

**Table 5:** Platinea’s 10 prioritized solutions addressing all areas of shortage causes (June 2020).

**0. Clarify the medical value of antibiotics (core principle stressing the need of new valuation models)**
1. **Security stocks** (centrally coordinated and increased inventory levels)
2. **Central coordination with a master-planner** (nationally responsible for drug availability, need monitoring and prioritization during shortages) 2.1. **Centralized tendering/purchasing**, but with **parallel, multiple suppliers selected**
3. **Integrated computer system with overview on stock levels; AI prognoses to balance needs and supplies:** cockpit for master-planner (showing alternative MAHs during shortages, bottlenecks, and supply chain’s risks, clearing mechanisms allocating deliveries between MAHs)
4. **Norwegian model:** rewards and/or supplier selection based on sustainability and delivery security (esp. supply chains with multiple, geographically spread FDF/API supplier registered): 4.1 **Inpatient care/hospital** (regional level) 4.2 **Outpatient care/community** (national auctions)
5. **Spreading supply volumes among several suppliers** in tendering procedures
6. **Increased max prices in auction system for products with very few MAHs** which risk deregistration
7. **Yearly fixed payment for generic antibiotics with volume commitments** (subscription model)
8. **Simplified regulatory processes** to introduce to Sweden old antibiotics approved in other countries
9. **MAHs supporting orderly withdrawals** with substitute supplier/MAH (incentive to transfer authorizations/product registrations and/or obligations to inform early MPA about withdrawal)
10. **Nordic collaboration for purchase and production of older antibiotics**, esp. narrow-spectrum child formulations, e.g., rationalized and optimized product ranges, harmonized pack sizes

## Data Availability

Data is unavailable due to privacy or ethical restrictions.
